# Analysis of the Effects of the River Network Structure and Urbanization on Waterlogging in High-Density Urban Areas—A Case Study of the Pudong New Area in Shanghai

**DOI:** 10.3390/ijerph16183306

**Published:** 2019-09-09

**Authors:** Song Liu, Mengnan Lin, Chunlin Li

**Affiliations:** 1 Landscape Architecture Department, College of Architecture and Urban Planning, Tongji University, Shanghai 200092, China; 2 CAS Key Laboratory of Forest Ecology and Management, Institute of Applied Ecology, Chinese Academy of Sciences, Shenyang 110016, China

**Keywords:** urbanization, surface water ratio, river density, impervious surface ratio, urban waterlogging, Pudong New Area

## Abstract

In the process of urbanization, high-intensity human activities have seriously disturbed the river networks, especially in the core urban areas of large cities. At present, a series of urban water environmental problems, such as urban waterlogging and non-point pollution, caused by damage to the river network structure and the decrease in surface permeability conditions in high-density urban areas have received widespread attention. In this study, the effects of the river network structure and urbanization on urban waterlogging were analyzed in the Pudong New Area by using the data of waterlogging sites on the Amap. The results showed that the average water surface ratio is 10.9%, the average river network density is 4.59 km/km^2^, and the comprehensive impervious ratio is 42.8%. From northwest to southeast, the impervious ratio of the Pudong New Area decreases gradually, and the water surface ratio and the river density increase gradually, while the areas with high waterlogging density are mainly concentrated in the northwest districts. The correlation coefficients indicate that the river network structure (−0.710 and −0.716) has a greater impact on waterlogging than urbanization (0.608) does. The current water surface ratio (10.9%) of the study area generally meets the requirements of the suitable water surface ratio (10.0%) in a rainfall return period of 50 years. However, the water surface ratio and the river density in about half of the districts did not meet the requirements of the suitable river network structure.

## 1. Introduction

Rapid urbanization increases the area of impervious surfaces, a major factor that affects urban hydrologic processes through increased surface runoff volume, increased runoff velocity, decreased time of concentration and decreased groundwater recharge [1,2,3,4]. The generation of excess direct runoff and increasing urban flooding occurs as a result of changes to the urban microhydrological process and the insufficient capacity of urban drainage systems. In China from 2008 to 2010, urban flooding occurred in 62% of 351 investigated cities, and more than three severe flooding events occurred annually in 137 cities [5]. Large volumes of direct runoff generally increase the frequency and severity of flooding, resulting in social and environmental issues, including traffic interruption, property loss, water pollution and disease [6,7]. Urban flooding has become a serious threat to the sustainable development of cities undergoing rapid urbanization, especially in China [8]. Previous research found that the annual average loss of residence damage because of waterlogging in Shanghai central urban area was about CNY 22.25 million [9].

The structure of a river network is one of the most fundamental components of basin geomorphology [10]. The river network structure, such as the total length, the river density, the branch ratio, the water surface ratio, and the river development coefficient, is related to its ability to adapt to environmental changes [11]. However, many rivers in cities have been replaced by built-up areas due to urban construction and human activities, which reduce the water surface ratio and the river network density, resulting in the degradation of river network structure and the decline in river network storage capacity. A previous study demonstrated that over 60% of the river channels in the world have undergone profound changes due to urbanization [12]. Yuan et al. (2006) studied the effect of urbanization on river structure development and found that the quantity of the water area and river structure decided the ability of flood proofing and absorption [13]. Ji et al. (2014) reported that the storage capacity of rivers decreases with the aggravation of the urbanization process, resulting in an increase in the risk of waterlogging [14].

The method of researching urban waterlogging can monitor the waterlogging point during rainstorms or use hydrological models to simulate, which often consumes much time and money. However, the simulation results may deviate from the real waterlogging situation. With the continuous enrichment of big data, some scholars have suggested the feasibility of using large data to assess the degree of urban waterlogging [15]. In this study, we selected the Pudong New Area (PNA) of Shanghai, a typical highly urbanized area with an abundant river network in the Yangtze River Delta, as the study area. Because of the difficulty of on-the-spot monitoring of urban waterlogging, we used “Waterlogging Amap (AutoNavi, Beijing, China)” data to analyze the urban waterlogging situation in the PNA. Many previous studies have focused on urban waterlogging causes, influencing factors, waterlogging simulation, waterlogging early warning and stormwater management practices [3,16,17,18,19,20,21], while few have focused on the effects of the river network structure on waterlogging. Urban waterlogging is affected by many factors, such as river network structure, topography, urban pipeline network construction, urbanization and so on. The change of DEM in PNA region is very small (slope is less than 2%), which indicates that the topography of this region has little effect on waterlogging. The data of Shanghai urban pipeline network is difficult to obtain. In this study, water surface ratio and river density are chosen to represent the river network structure, and impervious surface ratio represents the degree of urbanization. Therefore, the main objective of this study is to analyze the current situation of the river network structure and urbanization in each district of the PNA by using the water surface ratio, river density and impervious surface ratio and then calculate the suitable river network structure to help optimize the river network structure and reduce urban waterlogging.

## 2. Materials and Methods

### 2.1. Study Area and Data

**Figure 1:**
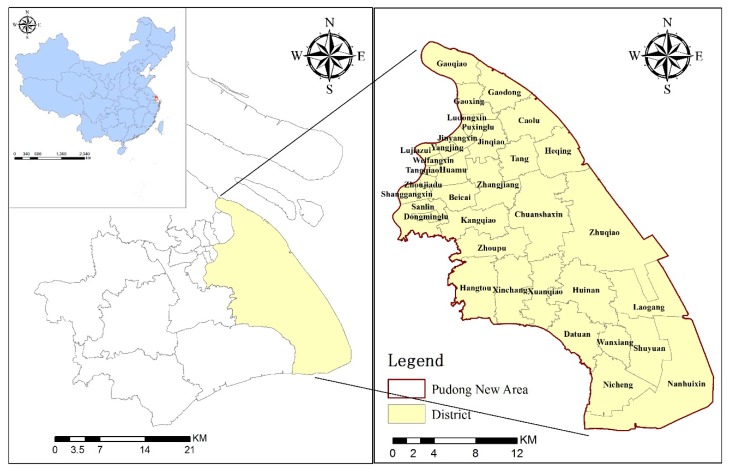
Regional location of the Pudong New Area (PNA) of Shanghai.

The PNA is located in the eastern part of Shanghai, between the estuary of the Yangtze River and the north beach of Hangzhou Bay (Figure 1) [22]. It is an eastern part of the Yangtze River Delta with an administrative area of 1379 km^2^ (Figure 1). There are 36 districts in the PNA, ranging from 3.7 to 160.2 km^2^. This area has a subtropical monsoon climate, with mild climate, abundant rainfall and distinct seasons. The average temperature is 17.6 °C, and the mean annual precipitation is 1207 mm. The maximum rainfall in the PNA was 1354.3 mm in 1957, and the minimum rainfall was only 657.7 mm in 1978, with a difference of 2.06 times. The distribution of rainfall is uneven during a year, mostly concentrated in May to October, accounting for more than 70% of the annual rainfall.

The PNA has undergone a remarkable transformation since the Chinese government formally announced plans for large-scale development here in 1990. The PNA has entered the process of rapid urbanization since then. It has developed from a typical land of fish and rice in the south of the Yangtze River Delta with a dense water network into a highly intensive urban area with an urbanization rate of 60.5% by 2017.

With the development of urbanization in the Pudong New Area, the urban impervious surface and building density increased, and the number, length and area of rivers have been declining year by year, especially when a large number of small rivers disappeared. According to the bulletin of the first national water conservancy census in Shanghai, from 1989 to 2010, the number of rivers in the former PNA (excluding Nanhui district) decreased by 4825, the length of the rivers decreased by 1415.45 km, and the area of the rivers decreased by 17 km^2^ [23]. At the same time, meteorological factors (plum rain, typhoon and extreme rainfall), natural factors (increasing impervious area, low-lying terrain) and social development factors (imperfect urban drainage) have jointly led to frequent waterlogging in the PNA.

**Figure 2:**
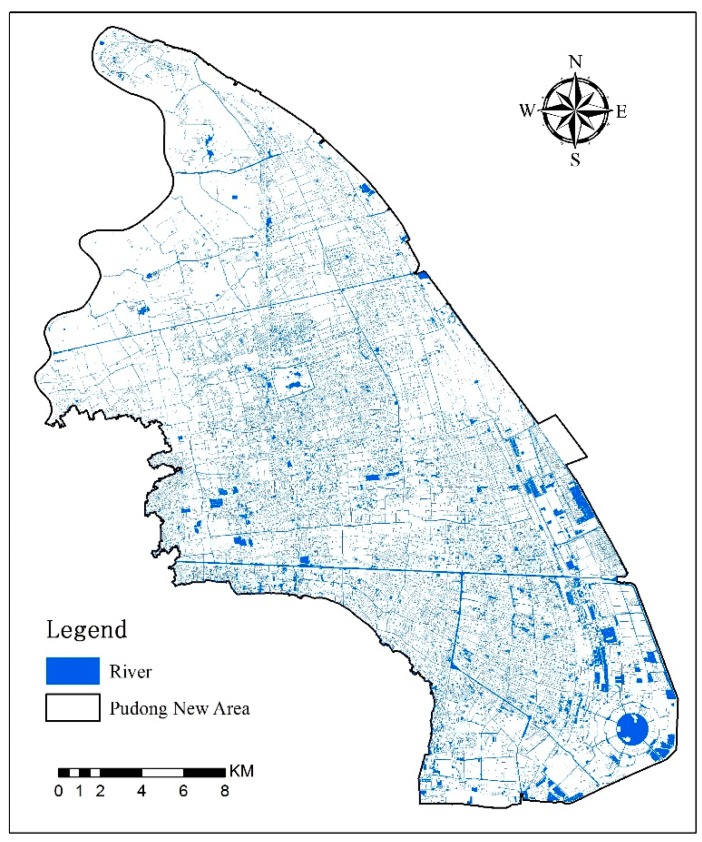
Distribution of the river system in the Pudong New Area.

Landsat 8 satellite images on August 2017 were used to interpret the impervious surface ratio in the PNA. The distribution map of the river system was interpreted on high-resolution images from Google Earth (Figure 2).

**Figure 3:**
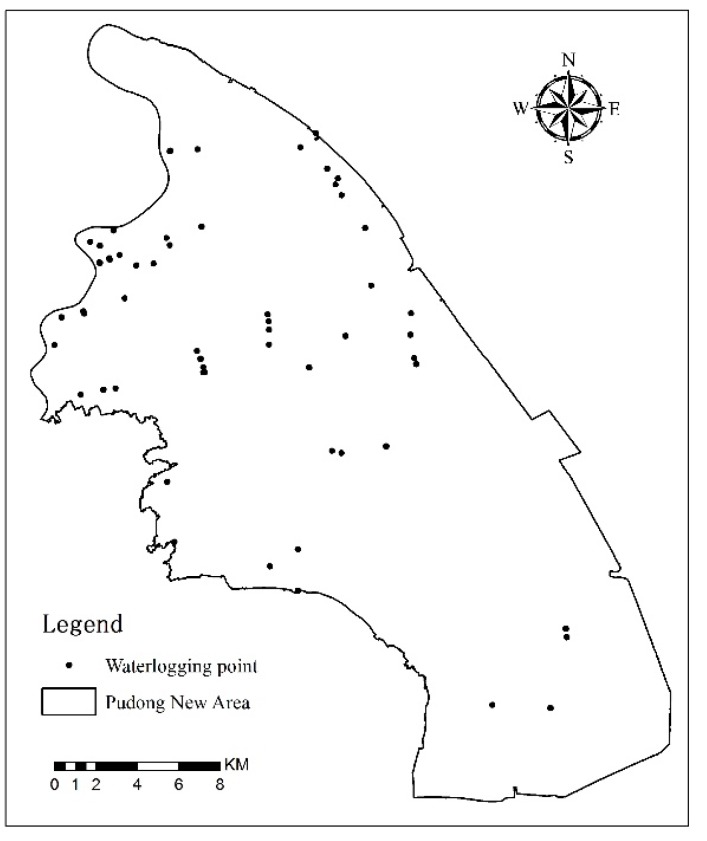
Distribution of the waterlogging points in the Pudong New Area.

The urban waterlogging map was developed by Amap (https://www.amap.com/), which can obtain the location and degree information of urban waterlogging points in the past five years based on historical data. Amap obtains information about road waterlogging locations in various ways, such as traffic police reporting, big data artificial intelligence calculation, flood prevention office field monitoring data, and user reporting. Finally, by synthesizing all the historical waterlogging data, the waterlogging points in this area are generated in the Amap app. In this study, we captured the location data of 61 waterlogging points in the PNA by Fiddler on Amap (Figure 3).

### 2.2. River Network Structure

The structure of the river network can be characterized using traditional statistical indicators, such as the total length, the river density [24], the branch ratio [25], the water surface ratio [11], the degree of curvature [26], and the self-similarity degree [27]. The water surface ratio and river density are common structural indexes used to characterize the water storage capacity of rivers. Among them, the water surface ratio is the basic index to ensure regional water security, while the river network density reflects the spatial level of regional drainage.

(1) $$D_{r}=L/F$$

(2) $$W_{p}=f/F$$

where *D_r_* is the river density, km/km^2^; *L* is the total river length, km; *F* is the total area, km^2^; *W_p_* is the water surface ratio; and *f* is the water surface area in the region, km^2^.

The total area of the water surface is calculated according to the river system distribution map of the PNA. The river network density is calculated by the weighted density method, that is, by calculating the ratio of the water surface area to the average weighted width of the river, and then divided by the total area of the study area.

### 2.3. Impervious Surface Ratio

Landsat 8 images on August 2017 were used to interpret the impervious surface ratio of each pixel by the linear spectral mixture analysis (LSMA) method [8,28]. Based on the vegetation-impervious surface-soil (V-I-S) model, each pixel of the urban land cover could be decomposed into a proportion of the vegetation, impervious surfaces, and soil [29]. To verify the accuracy of the proportion of the impervious surface ratio, we selected 100 random points with an area of 300 m×300 m. For each point, the vegetation, impervious surface and soil were visually interpreted on high-resolution images from Google Earth. Then, the root mean square error (RMSE) was computed to evaluate the difference between the LSMA interpreted values and the manually interpreted values. Through calculating, the RMSE of the impervious surface ratio was 0.21, which indicated that the accuracy of the impervious surface ratio interpretation by the LSMA was good enough for further analysis.

### 2.4. Suitable River Network Structure

The river network system is the main carrier for regional rainwater harvesting and utilization. The water surface ratio and river density in the region are comprehensive indicators reflecting the urban rainwater storage capacity and ecological carrying capacity. To determine the suitable river network structure, we must consider the influence of the rainfall characteristics, the underlying surface characteristics, the water storage capacity and other factors. Total rainfall runoff in a region is composed of land surface runoff and water surface runoff [30]:

(3) $$V=\frac{1}{1000}\left[P\times \left(F-f\right)\times K+P\times f\right]$$

where *V* is the total rainfall runoff in a region, m^3^; *P* is the rainfall, mm/h; and *K* is the regional composite runoff coefficient.

Usually, water level differences are used to characterize the hydrological storage capacity of river network systems as follows:

(4) ΔH=1000×V/f

where *ΔH* is the water level difference of a river network system, mm.

Using formulas 2, 3 and 4, we can obtain the formula for the water surface ratio and water level difference:

(5) $$W_{p}=\frac{K}{\frac{\Delta H}{P}-1+K}$$

According to the drainage and waterlogging prevention standard in the “Technical Guidelines for the Construction of Sponge Cities in Shanghai”, the waterlogging control standard in downtown Shanghai is to prevent large-area waterlogging caused by the rainfall of return period of 100 years, and other regions are rainfall of return period of 50 year [31]. Therefore, in this study, rainfall control objectives of 100-year and 50-year return periods are taken as guidance for a suitable range of water surface ratios. Based on the formula of short duration rainstorm intensity in Shanghai, the 100-year and 50-year return periods for rainfall in the PNA are 101 mm/h and 91 mm/h, respectively. According to the requirements of sponge cities, 70% of the rainfall must be absorbed and utilized locally in Shanghai [30]. Therefore, the 100-year and 50-year rainfall to calculate the suitable river network structure in this study is 70.7 mm/h and 63.7 mm/h, respectively. K is calculated by area weighted runoff coefficients of various land use, and the runoff coefficient of each land use is assigned according to the Technical Guide for Sponge Cities [30]. To ensure the safety of urban stormwater control, referring to the flood control planning of Shanghai, the water level difference of the river network system is taken as 500 mm [32]. The suitable range of river density is estimated by the weighted river width and suitable water surface ratio.

To reflect the suitability of the water surface ratio and river density, we introduced the water surface ratio index (*I_wsr_*) and the river density index (*I_rd_*).

(6) $$I_{wsr}=\frac{W_{p}-W_{sp}}{W_{sp}}$$

(7) $$I_{rd}=\frac{D_{r}-D_{sr}}{D_{sr}}$$

where *I_wsr_* is the water surface ratio index; *W_sp_* is the suitable water surface ratio; *I_rd_* is the river density index; and *D_sr_* is the suitable river density, km/km^2^. When the *I_wsr_* and *I_rd_* are negative, the river network structure is worse than the suitable river network structure. The smaller the value is, the larger the gap between the actual river network structure and the suitable river network structure. In contrast, when the *I_wsr_* and *I_rd_* are positive, the actual river network structure is better than the calculated suitable river network structure.

## 3. Results and Discussion

### 3.1. River Network Structure and Urbanization

**Figure 4:**
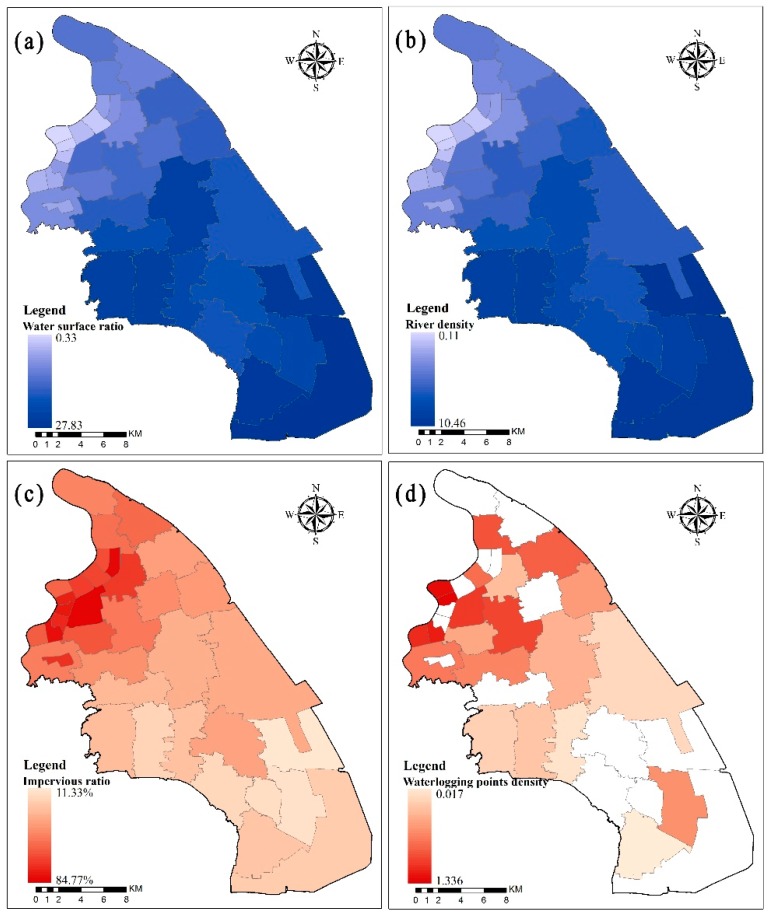
Characteristics of the Pudong New Area. (**a**) Water surface ratio of the Pudong New Area; (**b**) River density of the Pudong New Area; (**c**) Impervious ratio of the Pudong New Area; (**d**) Waterlogging points density of the Pudong New Area.

A distribution map of the river system was used to calculate the river network structure. In the PNA, the average water surface ratio is 10.9%, the average river network density is 4.59 km/km^2^, and the comprehensive impervious ratio is 42.8%. However, the spatial distribution of the water surface ratio, river density and impervious ratio in each district is obviously different. The water surface ratio and river density increased from northwest to southeast, while the impervious ratio decreased gradually from northwest to southeast (Figure 4a,b,c). In the northwest of the PNA, some districts with high urbanization levels, such as Lujiazui, Weifangxin, and Jinyangxin districts, are located within the inner ring line of Shanghai. With the process of urbanization, the green space rate in these districts has been reduced, and the small rivers have been decreasing, resulting in a relatively low water surface ratio and river density and a relatively high impervious ratio. In the southeast of the PNA, the urbanization level in these districts is low, the intensity of urban development is low, and the proportion of green land and farmland is larger, which leads to a relatively high water surface ratio and river density and a relatively low impervious ratio.

The density distribution of the waterlogging points shows that the spatial distribution of the waterlogging point is greater in the northwest and less in the southeast (Figure 4d). Many districts in the north of Shanghai, such as Weifangxin, Lujiazui, and Zhoujiadu, are located in the inner ring and near the Huangpu River. The water surface ratio and river density are low, and the impervious ratio is high in these districts, which results in increased runoff and poor drainage, further resulting in the high density of the waterlogging points. In contrast, in the southern part of the PNA, some districts with relatively low urbanization have low densities of waterlogging points, such as Niqiao, Xuanqiao and Hangtou.

### 3.2. Pearson’s Correlation Analysis

Based on the bivariate correlation analysis among the four factors of the water surface rate, river network density, impervious ratio and waterlogging point density, the relationships among the four factors of the 36 districts in the PNA were analyzed (Table 1). These results showed that the water surface ratio, impervious ratio, river density and waterlogging point density were significantly correlated at the 0.01 level. The Pearson’s correlation coefficients between the waterlogging point density and the water surface ratio, impervious ratio, and river density were −0.710, 0.608, and −0.716, respectively. The correlation coefficients indicated that the density of the river network has the greatest influence on the density of waterlogging, followed by the water surface ratio, and the impervious ratio has the smallest influence. Impervious surface is not only a key indicator of urbanization, but also a major contributor to the environmental impact of urbanization [7]. Our results illustrated that the river network structure has a greater impact on waterlogging than urbanization does. Many previous studies have shown that urbanization and river network structure can significantly affect water quality and quantity. Deng (2019) suggested strong correlations between the water quality and the structure and connectivity of the river network in the Southern Jiangsu Plain by using the grey relational analysis [11]. Deng and Xu (2018) found that actual flood regulation capacity exhibited a 33.2%-reduction with the development of urbanization during the 1960s to the 2010s [18]. Du et al. (2015) reported that urban waterlogging showed a very strong increasing trend because of insufficient capacity of urban drainage system and impacts of rapid urbanization in Shanghai from 1949 to 20019 by using a newspaper-based flood database [33]. Yang et al. (2016) found that the drainage density decreased by 20% and the water surface ratio decreased by 36%, which resulted in significant decreases in the storage and flood control capacities of the river networks [34].

River network structure is negatively correlated with waterlogging point density. The larger the water surface ratio and river network density are, the smaller the waterlogging degree, and the stronger the regulation capacity of the river is. There is a significant positive correlation between the impervious ratio and the waterlogging point density. The greater the impervious ratio is, the greater the waterlogging degree, and the weaker the regulation capacity of the rivers. At the same time, there are also significant correlations between the river network structure and the impervious ratio. Through correlation analysis, the correlation coefficients of the impervious ratio with the water surface ratio and river density are −0.881 and −0.907, respectively.

### 3.3. Suitable River Network Structure

According to the calculation of the suitable river network structure, the range of the suitable water surface ratio and the river density for the whole study area and the 36 districts were obtained. The current water surface ratio of the PNA is 10.9%, which is larger than the requirements of 10.0%. The current water surface ratio of the study area generally meets the requirements. However, according to the distribution of the water surface ratio in each district, only 18 districts met the standard, and half of the districts did not meet the requirements of the suitable water surface ratio.

**Figure 5:**
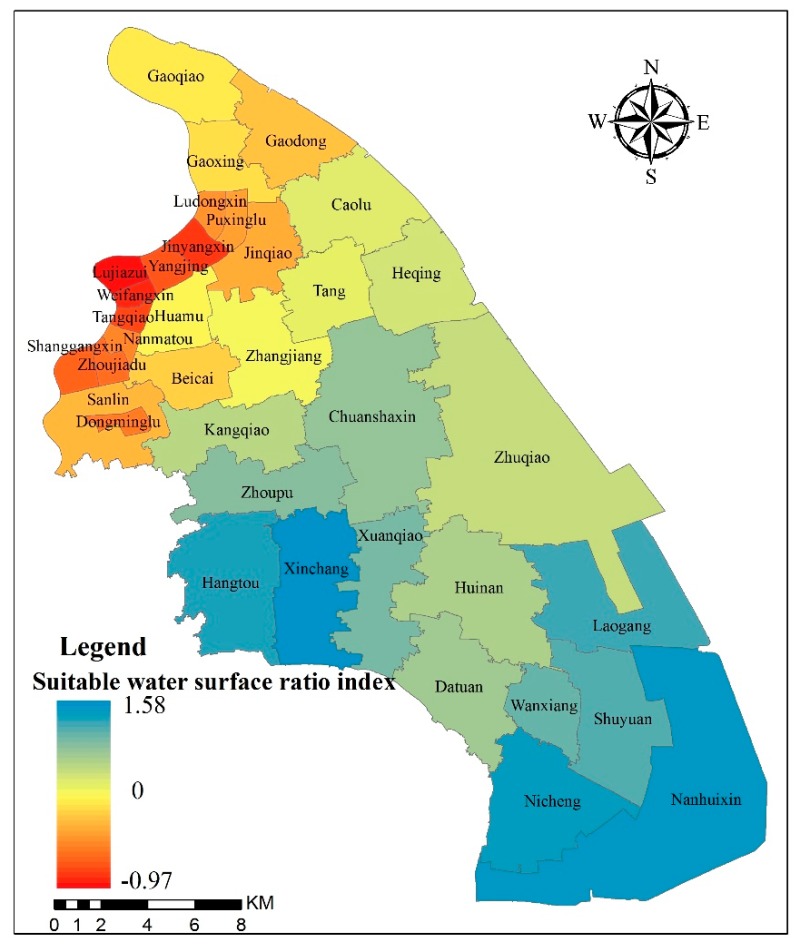
Suitable water surface ratio index of the Pudong New Area.

The *I_wsr_* of the PNA is 0.09, which revealed that, although the present water surface ratio in the whole study area is suitable, it is only slightly higher. Therefore, we should pay more attention to protecting the water surface of the PNA to prevent it from falling below the suitable water surface ratio. From the distribution of the water surface ratio index of each district, we can see that from north to south, the *I_wsr_* gradually increases, while the districts above the suitable water surface ratio are in the south and the districts below it are in the north (Figure 5). In the southern and southwestern districts of the PNA, the current water surface ratios of the Xinchang, Nanhuixin, Nicheng, Hangtou and Laogang districts are all above 20%, and the *I_wsr_* of these districts are the highest at 1.58, 1.57, 1.55, 1.49 and 1.42, respectively. In the northwestern districts of the PNA, the current water surface ratios of the Lujiazui, Weifangxin, Jinyangxin, Tangqiao and Yangjing districts are all under 2%, and the *I_wsr_* of the districts are the lowest at −0.97, −0.90, −0.88, −0.88 and −0.84, respectively.

**Figure 6:**
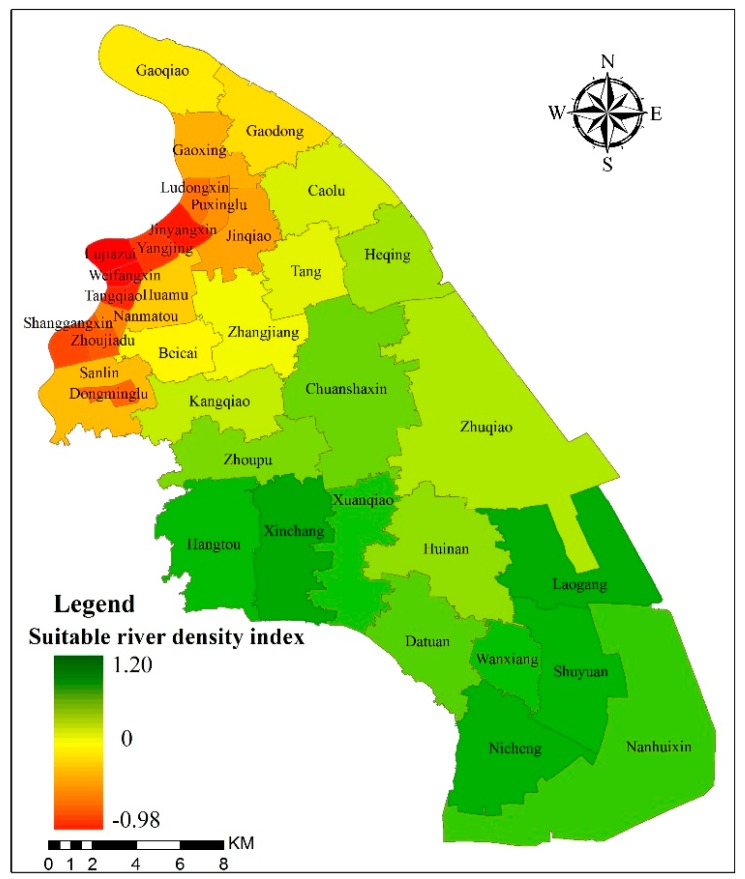
Suitable river density index of the Pudong New Area.

The current river density of the PNA is 4.6 km/km^2^, which does not reach the threshold of 5.1 km/km^2^ of suitable river density (Table 2). There are 19 districts meeting the requirements of suitable river density, accounting for 52.8%, and 17 districts that did not meet the suitable river density (Figure 6). The distribution of the suitable river density is basically the same as that of the suitable water surface ratio. Only the Zhangjiang district can reach the suitable water surface ratio but not the suitable river density. The distribution of the *I_rd_* in each district is similar to that of the *I_wsr_*. From northwest to southeast, the *I_rd_* increases gradually. The four districts with the worst suitable river density index are Lujiazui, Weifangxin, Jinyangxin and Tangqiao; their suitable river density is 6, 5.9, 6 and 5.9 based on the calculation, while the actual river density is 0.1, 0.6, 0.7 and 0.7, respectively.

There are significant differences in the spatial distribution of the water surface ratio and river density in the PNA, and the impact of urbanization is very significant. Therefore, there are great differences in the spatial distribution of the river system storage capacity, which may be the reason why waterlogging still occurs in some places of the PNA, although it achieves the suitable water surface ratio in the whole study area. And another major cause of local urban waterlogging is the different drainage pipelines capacity in each districts, which is unable to analyze its contribution due to lack of data. In some districts in the central and southern part of the PNA, the current water surface ratio and river density are much higher than the suitable water surface ratio and river density, which indicates that there is enough river system capacity to accept the return period of 50-year rainstorms, and the river system can fully meet the requirements of waterlogging prevention and control in the current urban situation. However, in some districts of the eastern PNA with relatively high urbanization, the gap between the current river network structure and the suitable river network structure is very large. The area and length of the river system are reduced, which indicates that the hydrological regulation capacity of the river system is seriously inadequate and far from meeting the requirements of urban waterlogging prevention and control. Therefore, it is necessary to regulate the regional flood control capacity by controlling the water surface ratio and river density of each district. For the districts that already meet the standards, it is necessary to ensure that the water surface ratio and river density do not decrease. For the districts that do not meet the standards, the flood regulation capacity of the river system can be improved by increasing the water surface ratio and river density in the renovation of the old city.

## 4. Conclusions

By calculating and analyzing the impact of the river network structure and urbanization on urban waterlogging, we proposed the suitable water surface ratio and river density to provide a scientific basis for the protection and control of river systems and the mitigation of urban waterlogging in each district. The following conclusions can be summarized as follows:

(1) In the PNA, the average water surface ratio is 10.9%, the average river network density is 4.59 km/km^2^, and the comprehensive impervious ratio is 42.8%. From northwest to southeast, the impervious ratio of the PNA decreases gradually, the water surface ratio and the river density increase gradually, while the areas with high waterlogging density are mainly concentrated in the northwest districts.

(2) The correlation coefficients indicated that the river network structure has a greater impact on waterlogging than urbanization does. The Pearson’s correlation coefficients between the waterlogging point density and the water surface ratio, impervious ratio, and river density were -0.710, 0.608, and −0.716, respectively.

(3) The current water surface ratio (10.9%) of the study area generally meets the requirements of the suitable water surface ratio (10.0%) in a rainfall return period of 50 years. However, the water surface ratio and the river density in about half of the districts did not meet the requirements of the suitable river network structure. The distribution of the *I_wsr_* and *I_rd_* varied greatly in each district but increased gradually from northwest to south.

In this study, the waterlogging points data are transformed into the waterlogging point density to reflect the degree of waterlogging. Compared with the traditional long-term monitoring data, the accuracy and comprehensiveness may be insufficient under the condition of less data accumulation. With the continuous enrichment of data, monitoring point data or simulation data can improve the reliability of the analysis results. The suitable river network structure index we proposed in this study is the most innovative point, which is a good method for evaluating the rainfall-flood capacity of river systems and helps to optimize the river system structure and alleviate urban waterlogging. However, there still been some limitations in terms of more general application that we should to pay special attention to. The regional composite runoff coefficient K we calculated in this study is based on the Technical Guide for Sponge Cities, which only recommended runoff coefficients of different land use types for Chinese cities and may not be suitable for other regions. Therefore, the regional composite runoff coefficient needs to be calculated and calibrated according to the real conditions of study area for further application.

## Figures and Tables

**Table 1:** Pearson’s correlation among the four factors.

Variables	Water Surface Ratio (%)	Impervious Ratio (%)	River Density (km/km²)	Waterlogging Points Density (unit/km²)
Water Surface Ratio (%)	1	−0.881 ^**^	0.988 ^**^	−0.710 ^**^
Impervious Ratio (%)	-	1	−0.907 ^**^	0.608 ^**^
River Density (km/km²)	-	-	1	−0.716 ^**^
Waterlogging Points Density (unit/km²)	-	-	-	1

^**^ Correlation is significant at the 0.01 level (2-tailed).

**Table 2:** Suitable river network structure of the Pudong New Area.

Districts	Water Surface Ratio (%)	Suitable Water Surface Ratio (%)	*I_wsr_*	River Density (km/km²)	Suitable River Density (km/km²)	*I_rd_*
Beicai	8.4	11.0	−0.24	4.0	5.6	−0.29
Caolu	11.8	9.2	0.28	4.8	4.7	0.02
Chuanshaxin	18.3	8.8	1.08	7.6	4.5	0.69
Datuan	15.8	8.1	0.95	7.3	4.1	0.78
Dongminglu	3.4	11.0	−0.69	1.7	5.6	−0.70
Gaodong	7.2	9.9	−0.27	2.9	5.0	−0.42
Gaoxing	8.3	10.2	−0.19	2.7	5.2	−0.48
Gaoqiao	8.5	10.1	−0.16	3.1	5.2	−0.40
Hangtou	20.9	8.4	1.49	8.6	4.3	1.00
Heqing	12.6	9.4	0.34	5.6	4.8	0.17
Hudongxin	3.8	11.6	−0.67	1.9	5.9	−0.68
Huamu	11.1	11.5	−0.03	3.4	5.9	−0.42
Huinan	16.0	8.9	0.80	6.6	4.6	0.43
Jinqiao	6.5	10.8	−0.40	2.6	5.5	−0.53
Jinyangxin	1.4	11.7	-0.88	0.7	6.0	−0.88
Kangqiao	12.8	9.0	0.42	4.8	4.6	0.04
Laogang	24.0	9.9	1.42	10.5	5.0	1.10
Lujiazui	0.3	11.7	−0.97	0.1	6.0	−0.98
Nanhuixin	27.8	10.8	1.57	10.0	5.5	0.82
Nanmatou	3.8	11.6	−0.67	1.9	5.9	−0.68
Nicheng	21.4	8.4	1.55	9.0	4.3	1.09
Puxinglu	4.1	11.4	−0.64	1.9	5.8	−0.67
Sanlin	6.2	10.1	−0.39	2.8	5.1	−0.45
Shanggangxin	2.4	11.2	−0.79	1.0	5.7	−0.82
Luyuan	18.2	8.1	1.25	8.4	4.1	1.05
Tang	11.1	9.3	0.19	4.8	4.7	0.02
Tangqiao	1.4	11.6	−0.88	0.7	5.9	−0.88
Wanxiang	17.7	8.2	1.16	8.1	4.2	0.93
Weifangxin	1.2	11.6	−0.90	0.6	5.9	−0.90
Xinchang	20.9	8.1	1.58	9.0	4.1	1.20
Xuanqiao	18.0	8.4	1.14	8.1	4.3	0.88
Yangjing	1.9	11.6	−0.84	1.0	5.9	−0.83
Zhangjiang	11.7	10.3	0.14	5.1	5.2	−0.02
Zhoujiadu	3.0	11.4	−0.74	1.6	5.8	−0.72
Zhoupu	17.5	8.3	1.11	6.9	4.3	0.60
Zhuqiao	12.9	9.3	0.39	5.4	4.7	0.15
Pudong New Area	10.9	10.0	0.09	4.6	5.1	−0.10
